# P-2149. Cryptococcosis in Patients with Hematologic Malignancy: Guideline-Based Treatment Regimens and Mortality of a 30-year Cohort at Duke

**DOI:** 10.1093/ofid/ofaf695.2312

**Published:** 2026-01-11

**Authors:** Matthew L Goodwin, Julia A Messina, John R Perfect, Marhiah Montoya, Charles D Giamberardino, Andrea Sitlinger, Danielle Brander

**Affiliations:** Duke, Durham, NC; Duke University, Apex, North Carolina; Duke University, Apex, North Carolina; Duke, Durham, NC; Duke University, Apex, North Carolina; Duke University, Apex, North Carolina; Duke, Durham, NC

## Abstract

**Background:**

Cryptococcosis is categorized within 3 at-risk populations: HIV-infected, solid organ transplant, and HIV-negative/non-transplant. Despite uniform treatment recommendations among these diverse populations, the distribution of immunocompromising conditions of infected patients has changed dramatically over the past 30 years. The present study characterizes infection severity, treatment, and mortality in patients with hematologic malignancies, a population previously identified as having 100% 2-year all-cause mortality (Kaplan, *et al*. *Cancer*. 1977).

**Table 1.**
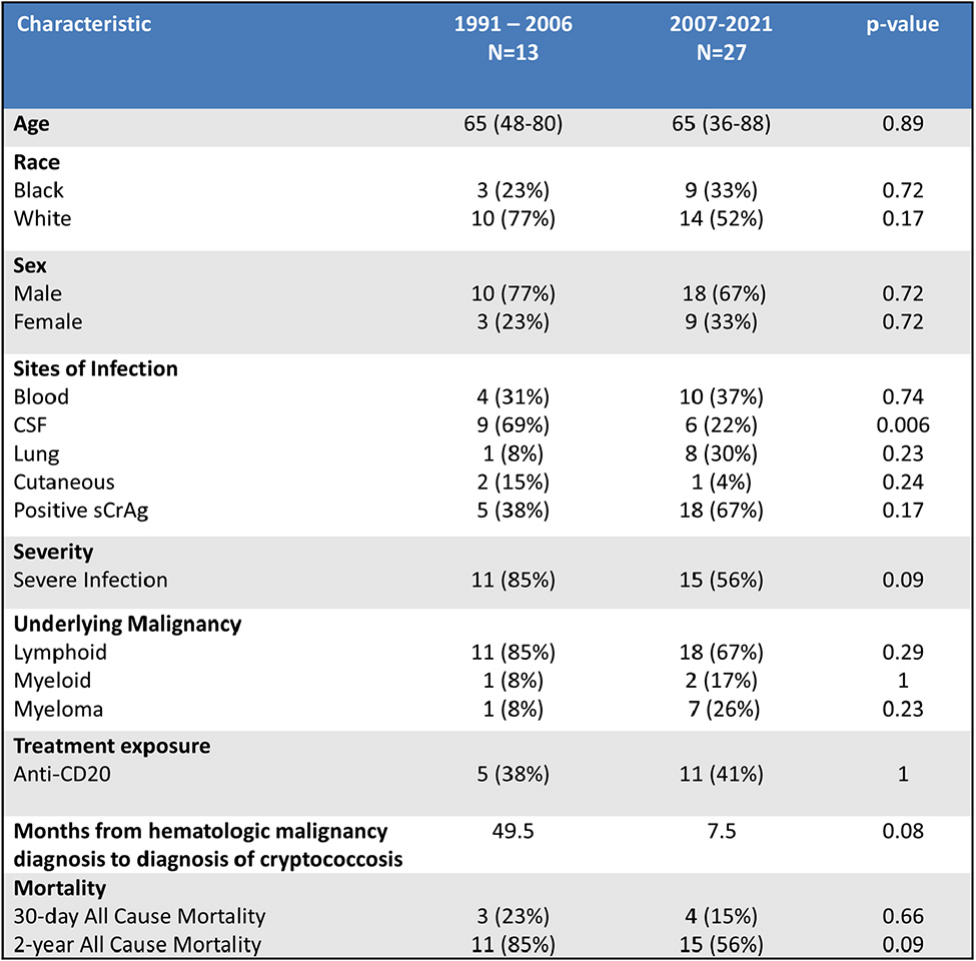
Patient demographics

**Table 2.**
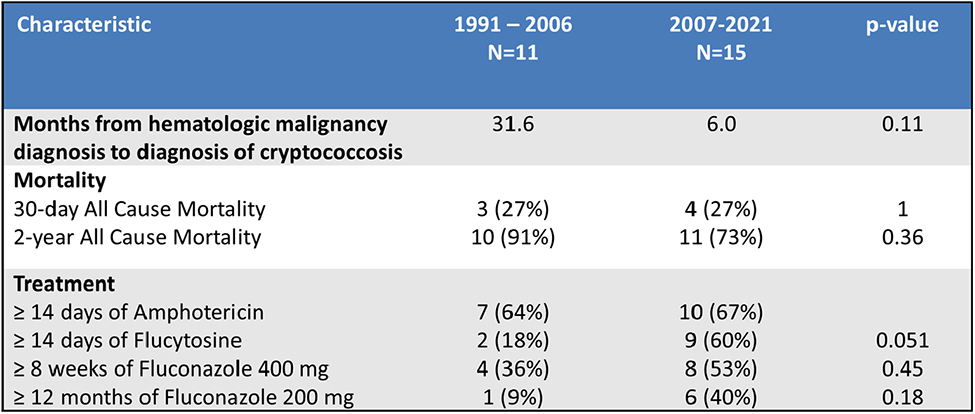
Treatment and outcomes of patients with severe cryptococcosis

**Methods:**

This is a cohort of 46 patients diagnosed with cryptococcosis by positive cultures, cryptococcal cerebrospinal fluid (CSF) or serum antigen, or histopathology at Duke from 1/1/91-12/31/21. Severe disease was defined as *Cryptococcus* present in CSF or blood cultures or serum antigen titer >1:512. True infection was defined as a positive diagnostic test that prompted antifungal treatment. Induction, consolidation, and maintenance treatment were defined by IDSA and ECCM guidelines. We then compared patients’ treatment regimens and mortality from the first 15 years of the study to the second 15 years.

**Figure 1.**
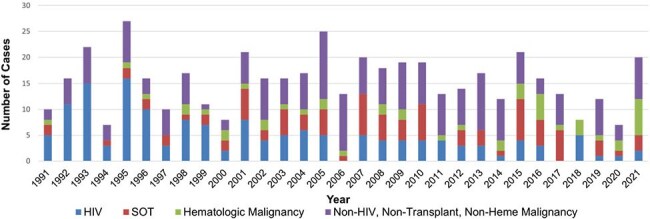
Annual cases of cryptococcus by underlying condition

**Figure:**
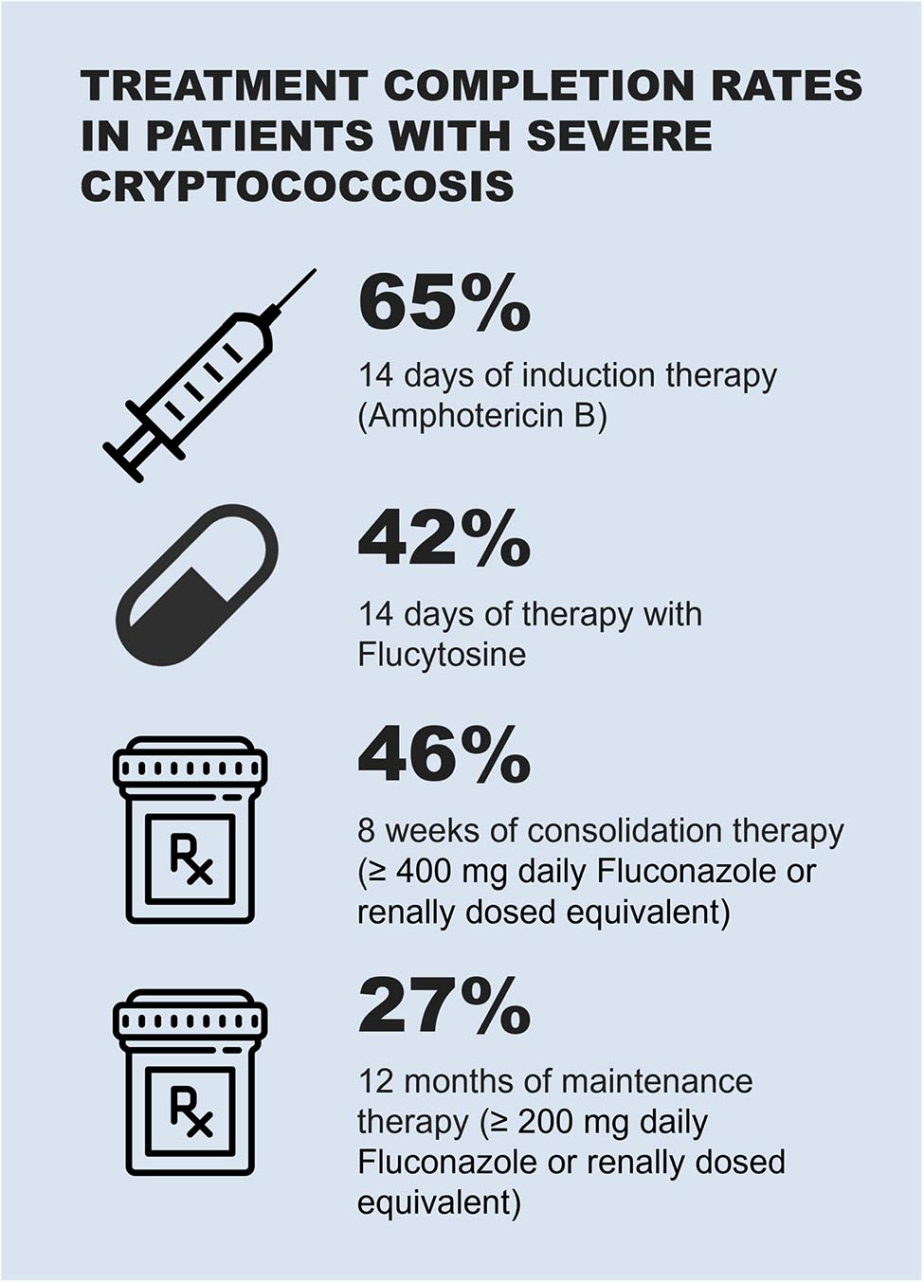

**Results:**

Of 46 patients identified, 40 patients had true infection and treatment records available. 65% presented with severe disease. Among patients with severe disease, in the first 15 years of our study compared to the second 15 years 18% vs. 60% completed a 2-week induction course of both amphotericin and flucytosine, 36% vs. 53% completed consolidation with 8 weeks of fluconazole ≥400 mg, and 9% vs. 40% completed maintenance therapy with 12 months of fluconazole ≥200 mg daily. 2-year all-cause mortality in the first 15 years was 91% vs. 73% in the second 15 years.

**Conclusion:**

During the 30-year study period, there was improvement in administration of guideline-based antifungal therapy when comparing patients with severe infection during the first half of the study to the second half. However, two-year mortality for patients with hematologic malignancies and cryptococcosis remains high, necessitating a better understanding of diagnostic and treatment strategies given significant comorbidity with concurrent cancer treatments.

**Disclosures:**

Julia A. Messina, MD, MHS MS, Seres: Advisor/Consultant|UpToDate: Royalties Andrea Sitlinger, MD, BeiGene: Advisor/Consultant|DAVA Oncology: Honoraria|Genmab: Grant/Research Support|Loxo Oncology: Grant/Research Support

